# Case Report: Home initiation of nocturnal non-invasive ventilation in two adolescents with Duchenne muscular dystrophy and comorbid autism spectrum disorder and ADHD

**DOI:** 10.3389/fped.2025.1525365

**Published:** 2025-03-14

**Authors:** Pien M. M. Weerkamp, Mark Voermans, Moniek Finders, Arno Brouwers, Philippe Collin, Sylvia Klinkenberg, Jos. G. M. Hendriksen

**Affiliations:** ^1^ Kempenhaeghe, Department Centre for Neurological Learning and Developmental Disabilities, Heeze, Netherlands; ^2^ Department of Pulmonary Diseases/Home Mechanical Ventilation, Maastricht University Medical Centre, Maastricht, Netherlands; ^3^ Koraal Gastenhof, Sittard, Netherlands; ^4^ Department of Neurology, Maastricht University Medical Centre, Maastricht, Netherlands; ^5^ Faculty of Health, Medicine and Life Sciences, School for Mental Health and Neuroscience, Maastricht University, Maastricht, Netherlands; ^6^ Duchenne Center, Heeze, Netherlands

**Keywords:** Duchenne muscular dystrophy, nocturnal non-Invasive ventilation, neuropsychiatric and neurocognitive comorbidities, psychological intervention, psychopharmacological intervention, case report

## Abstract

This case report describes initiation of Nocturnal Non-Invasive Ventilation in home settings for two adolescents with Duchenne Muscular Dystrophy and different neuropsychiatric and neurocognitive comorbidities: one has Autism Spectrum Disorder, and the other has Attention Deficit Hyperactivity Disorder, obsessive thinking and anxiety. This report emphasizes the need for a multidisciplinary team approach, incorporating medical, psychological and psychopharmacological interventions for successful Non-Invasive Ventilation implementation. Challenges include recognition of neuropsychiatric and neurocognitive comorbidities often seen in Duchenne Muscular Dystrophy. Lessons learned from these cases underscore the importance of: coordinated multidisciplinary efforts, early advanced care planning, accurate psychoeducation, cautious psychopharmacological interventions, and parental and patient involvement. To our knowledge, this is the first case report describing implementing Nocturnal Non-Invasive Ventilation in home settings in adolescents with Duchenne Muscular Dystrophy and neuropsychiatric and neurocognitive comorbidities. In conclusion, implementation at home can be successful and become a realistic target for each individual with Duchenne Muscular dystrophy.

## Introduction

Duchenne Muscular Dystrophy (DMD) is a neuromuscular disorder characterized by the dysfunction of the dystrophin protein which results in a spectrum of severe clinical manifestations, including delayed motor milestones, the eventual loss of independent ambulation, and the development of life limiting cardiac and respiratory complications (1). In line with the standards of care (2), mechanical ventilatory support becomes an essential intervention during the second to third decade of the patient's life. Initially, this support is introduced to address sleep-related breathing disorders and hypoventilation and is thereby important for improvement of quality of life as e.g., fatigue may play a serious debilitating role in daily functioning. As the disease progresses Non-Invasive Ventilation (NIV) becomes necessary, as respiratory muscles further deteriorate. The progression of the disease eventually leads to a stage of full dependence on mechanical ventilation.

The profound impact of ventilatory support on DMD patients survival is noteworthy, extending life expectancy from 19 to 27 years (3). These results suggest that NIV contributes to improved survival in DMD, though outcomes may vary depending on disease progression, comorbidities, and adherence to ventilation therapy. Nonetheless, the results are promising. However, limited data exists regarding NIV adherence among (pediatric) patients with neuromuscular disorders. Only a few studies report on this (4–8). A recent study on NIV adherence in adolescents with DMD has indicated that internalizing problems (e.g., depression and anxiety) may cause challenges (5). Notably, the risk of neuropsychiatric comorbidities in DMD is higher than in the general population [see Pascual-Morena et al. (9) for a systematic review and meta-analysis]. The overall prevalence of Autism Spectrum Disorder (ASD), Attention Deficit Hyperactivity Disorder (ADHD), anxiety disorders and obsessive compulsive disorders are 7.0%, 18.0%, 24.0%, and 12% respectively, underscoring the significance of addressing these disorders in the context of NIV at home.

In this context, we describe two patients that shed light on the specific – nonphysical - psychological challenges faced by DMD adolescents when introducing home ventilation. Aim of this case report is to describe the interplay between behavioral factors and (nocturnal) NIV usage, and identify potential psychological and psychopharmacological strategies for successful NIV implementation at home. To our knowledge, this is the first case report of two adolescents with DMD with neuropsychiatric and neurocognitive comorbidities where NIV is initiated in the home situation.

## Patient information

### Case 1

A sixteen-year-old male adolescent diagnosed with DMD (deletion of exon 46–50 in the dystrophin gene) and comorbid ASD diagnosis [based on the Diagnostic and Statistical Manual of Mental Disorders fifth edition (10)]. His medical history indicates the administration of on intermittent prednisolone regimen, periodic use of melatonin ante noctem and in the past he (effectively) used Risperdal (0.5 mg) for a short period of time.

A structured neuropsychological assessment was conducted at the age of 13.5 showing a disharmonic profile with strong visual thinking (nonverbal concept formation) and weak verbal capacities (receptive and expressive). See Appendix 1 (Table 4) for an overview. Assessment of his respiratory function before the initiation of nocturnal home ventilation showed that the criteria for NIV implementation were met (see Table 1). According to the 2021 Dutch Guidelines on Chronic Ventilation (11), NIV is recommended for neuromuscular patients when pCO₂ or transcutaneous CO₂ exceeds 6.0 kPa, Forced Vital Capacity (FVC) falls below 50%, or hypoventilation-related symptoms are present. The NIV settings were titrated based on nocturnal transcutaneous CO2 measurements and data retrieved from the ventilator, with particular attention given to tidal volumes and respiratory frequency. These measurements were performed in a home setting.

**Table 1:** Assessment of respiratory function in two adolescents with DMD.

Function test		Case 1	Case 2
Transcutaneous CO_2_^a^	tCO_2_	6.43 kPa (range 5.75–6.94)	6.15 kPa (range 5.56–6.47)
		48.2 mmHg (range 43.1–52.0)	46.1 mmHg (range 41.7–48.5)
Forced vital capacity	FVC	54% predicted value	56% predicted value
Highest peak cough flow	PCF	160 L/min^b^	240 L/min^b^

kPa, kilopascal; L/min, liters per minute; mmHg, millimeters of mercury.

^a^ Obtained overnight using Sentec transcutaneous monitoring system at home.

^b^ Value <270 L/min indicates significantly diminished cough function.

In view of previous reported serious behavioral problems with novel situations (change of bedroom) in combination with the diagnosed ASD, it was decided that initiating NIV in a pediatric ICU environment was not realistic. In contrast to adult care (12, 13), initiating nocturnal ventilation at home has not yet been applied in children in the Netherlands. A 12-month timeframe was set. The main intervention strategies were, initially, putting a multidisciplinary team together including: his parents, a pediatric intensivist and specialized nurse (both from the department of home ventilation), a child clinical psychologist, child neurologist, child psychiatrist, and from school (special education) a nurse, speech therapist, and teacher (see Table 2 for further elaboration on the responsibilities per team role). Secondly, the best possible preparation for using and getting acquainted to the ventilator was communicated through visual support (pictograms). Furthermore, it was determined with his parents prior to his implementation when and what to share with the patient, managing the flow of information, timing, and extent of patient involvement. It was also considered important to create a detailed schedule which maintained the “normal” structure in the home environment according to the - Treatment and Education of Autistic and related Communication handicapped Children (TEACCH)-principle (14). Last of all, psychopharmacological treatment (Risperdal start 0.5 mg/day, max. 1.0 mg/day) was initiated weeks before start of home ventilation. With the expectation that it would be effective again, as it had been previously and its evidence in literature (15). Following this preparation he accepted the ventilation without any problems from the second night on. The psychopharmacological treatment was successfully tapered down after some weeks.

**Table 2:** Responsibilities in a multidisciplinary team with current and possible team roles.

Responsibilities in our multidisciplinary team	Current assigned roles in our team	Other possible team roles
Providing primary care, legal representation, and emotional support to the child	Parents	Legal guardians, caregivers
Ensuring structured routines and communication tailored to the child's cognitive and emotional needs	Parents, speech therapist, child psychologist, teacher	Caregivers, psychotherapist, nurse (practitioner), teacher
Monitoring respiratory function and ensuring effective home ventilation management	Nurse (from home ventilation center), Intensive Care specialist	(Child) Pulmonogist
Assessing cognitive, emotional, and behavioral challenges related to DMD	Child psychologist, child neurologist	Psychiatrist
Developing communication strategies for children with limited verbal abilities	Speech therapist, child psychologist, parents, teacher	–
Managing psychiatric symptoms and prescribing psychofarmaca if necessary	Child psychiatrist, child neurologist	Psychiatrist
Coordinating respiratory interventions, including non-invasive ventilation initiation	Intensive Care specialist, nurse (from home ventilation center)	Home ventilation specialist, pulmonologist
Implementing airway clearance techniques such as air stacking	Nurse (from home ventilation center), parents	Respiratory therapist, rehabilitation specialist
Coordinating multidisciplinary care to ensure a holistic approach	Intensive Care specialist, child neurologist, child psychologist	Multidisciplinary home care teams
Addressing ethical considerations and family decision-making in treatment planning	Child psychologist, child neurologist, nurse (from home ventilation center), parents	Medical social worker, general practitioner
Providing physical advice or therapy support to optimize lung function and mobility	Nurse (from home ventilation center), physiotherapist	Rehabilitation specialist

Within two months of starting NIV, the patient exhibited a noticeable improvement in sleep patterns, as reported by the parents. The adolescent woke up feeling more rested and happier compared to before the treatment. Furthermore, the parents also reported positive effects on the adolescent's overall development.

### Case 2

A seventeen-year-old male adolescent diagnosed with DMD (deletion of exon 49–50 in the dystrophin gene) and a comorbid diagnosis of ADHD (based on DSM-5 criteria).

At the age of thirteen, a structured neuropsychological assessment indicated average intelligence scores, with below-average performance in working memory and a low score in arithmetic (see Appendix 1 for an overview). At the age of 15 he experienced a disrupted sleep pattern and increasing fatigue. Based on the combination of clinical symptoms and tCO_2_ measurement (see Table 1 for the results of respiratory assessment) the criteria for NIV implementation were met. In accordance with the normal procedure this was attempted at the age of 15 on the pediatric ICU of our hospital. This first attempt was unsuccessful due to: (1) the inadequacy in empowering the patient to make well-informed decisions, (2) insufficient detailed information -concerning home ventilation in daily practice, (3) insufficiently detailed time plan regarding initiation of ventilation, and (4) too much perceived pressure from the team on the patient to make it successful in a clinical setting.

Considering his unsuccessful and traumatic hospital experiences, a decision was made to attempt initiating NIV at home at age 17. Collaborating with the adolescent, his parents, and the same teams from the department of home ventilation and Kempenhaeghe (see Table 2) a plan was developed. Stress reduction techniques using mindfulness and hypnotherapy, EMDR-like approaches, as well as cognitive behavior therapy interventions (helpful thinking) were implemented, acknowledging that breathing exercises during NIV might not be effective.

After nine months a new attempt was made to set up NIV at home. Additionally, treatment with Fluoxetine was initiated because of obsessive thoughts and worrying because of his earlier experiences with NIV. This led to diminishing of obsessive thoughts and significant improvement of his mood. He gradually adjusted to the face mask and could relax while using it, even managing short periods of sleep. However, a viral respiratory infection disrupted progress despite his determination. Initially, he decided in consultation with our team to make a new attempt a few months later. At that moment however he indicated that he understood what benefits the treatment could bring him but that stress related to the ventilation had the upper hand. He decided not to start the treatment again and choose quality of life instead of quantity of life.

In Table 3 we summarize our lessons learned from both cases and may offer a guideline for other clinicians.

**Table 3:** Lessons learned from Two boys with duchenne muscular dystrophy and brain related comorbidities requiring home ventilation.

Topic	Lessons learned
Multidisciplinary approach	Coordination among involved specialists (child neurologists, respiratory therapists, psychologists, nurses, psychiatrist in consultation) is essential.
	Regular team meetings enhance communication and alignment of treatment strategies, considering both medical and behavioral aspects.
Early advanced care planning	Initiate advanced care planning early to allow individuals to consider and communicate their healthcare preferences, values, and treatment goals.
	Open discussions with healthcare providers, parents, and patients, enabling informed decision-making and ensuring that individual healthcare wishes are known and respected.
Psychoeducation	Providing psycho-education to the family about the implications of home ventilation is essential.
	Use clear language and visual aids to communicate complex medical information to adolescents and parents/caregivers
Preparation	Develop a detailed care plan with clear instructions for emergencies and daily care tasks.
Psychopharmacological treatment	Carefully weigh the risks and benefits of psychopharmacological interventions, with regular monitoring [see (16)].
Parental involvement	Acknowledge the crucial role parents have in the care and management of their child. They know their child best.
Ventilation equipment	There are different types of NIV masks and mouthpieces. Tailor the choice to individual comfort and needs.
	Gradual introduction and adaptation to the sound of the ventilation machine.
Respecting child's pace and autonomy	Respect the pace and autonomy of the child during treatment, allowing for gradual adjustment to new routines and interventions.
	Involve the child in decision-making when appropriate, empowering them to take ownership of aspects of their care and to set their personal limits
Knowledge of home situation	Gain comprehensive knowledge of the home environment.−Tailor treatment plans to align with the routines and dynamics of the specific home situation.

## Discussion

These cases show that home NIV coordinated by a multidisciplinary team may be a safe and important option to consider, especially when neuropsychiatric and neurocognitive comorbidities play a role in daily functioning. The combination of preparation, environmental structuring, psycho-education, and psychopharmacological treatment is essential. The big advantage of initiating this treatment at home is the embedding of the NIV in the daily structure and routine of the safe home environment including more autonomy for the patient. In the second case we were unable to initiate NIV at home during the first attempt due to acute medical circumstances. Furthermore, the distressing experiences of his attempt in the hospital setting and the interfering viral respiratory infection disrupted progress, which left him unable to start home NIV again for the moment. However, these experiences gave him an insight on the importance of quality of life and gaining more autonomy in decision making.

These results align with previous research, notably the study conducted by Pascoe et al. (5), highlighting the significance of a multidisciplinary approach in promoting NIV adherence in DMD and addressing the negative impact of internalized behavioral issues, such as anxiety and depression. A previous case study described a 20 year old male with DMD, who refused Positive Airway Pressure therapy due to claustrophobia and panic attacks in. This lead to admission to the emergency room with acute neurological symptoms. Also, this case emphasizes the importance offering essential psychological support to DMD patients (6) during life-changing events.

In light of the findings presented in these cases, it is concluded that a combination of careful preparation and psychological treatment holds promise in facilitating the implementation of home NIV in adolescents with DMD and comorbid neurodevelopmental or (internalized) behavioral symptoms. As such, it is strongly recommended to adopt a multidisciplinary timely approach when managing such cases, with particular attention to identifying and addressing potential psychological challenges, in order to optimize the support of the physical course of DMD. The possibility of nocturnal NIV should be discussed well in advance and a plan should be drawn up and discussed to provide adequate information to empower patients to make an informed decision together with their parents.

## Data Availability

The datasets for this article are not publicly available due to concerns regarding participant/patient anonymity. Requests to access the datasets should be directed to the corresponding author.

## Appendix 1

**Table 4:** Neurocognitive outcomes and brain related comorbidities of the two cases.

Neurocognitive and neuropsychiatric domains	Neurocognitive and neuropsychiatric subdomains	Case 1	Case 2
Intelligence	Non-verbal intelligence	Normal	Normal
	Verbal intelligence	Disturbed	Normal
Cognitive functions	Working memory		
	- Digit span	Disturbed	Normal
	- Sequential		Normal
	Executive functioning		
	- Planning		Normal
	- Attention		Normal
	Visual information processing	Normal	Normal
Academics	Speeded word reading		Normal
	Speeded arithmetic		Disturbed
Brain related comorbidities	Autism spectrum disorder	Diagnosis	
	Attention hyperactivity deficit disorder		Diagnosis
	Anxiety		Symptoms
	Obsessive compulsive disorder		Symptoms

Scores are grouped into two categories – normal and disturbed – based on their deviation (±2 SD) from the age appropriate mean. Brain related comorbidities are also grouped into two categories: he was diagnosed by psychiatrist or psychologist or he exhibits indicative symptoms.

Academics; **speeded arithmetic** = Tempo Test Automatiseren (TTA), **speeded word reading** = Continu Benoemen & Woordleze (CB&WL), Cognitive functions; **executive functioning** = Mazes of Wechsler Intelligence Scale for Children-third edition (WISC-III), **visual information** and **working memory** = Kaufmann ABC (KABC-II), Intelligence (in case 2); Wechsler Intelligence Scale for Children-fifth edition WISC-V, **nonverbal-IQ** (in case 1) = Raven's Progressive Matrices 2 and Snijders-Oomen nonverbal intelligence test (SON-R 6-40), **verbal IQ** (in case 1) = Peabody Picture Vocabulary Test (PPVT-III).
